# Supplementary material for: Early downregulation of hair cell (HC)-specific genes in the vestibular sensory epithelium during chronic ototoxicity
Source: J Biomed Sci. 2025 Sep 4;32:84. doi: 10.1186/s12929-025-01180-4 (PMC12409954; doi:10.1186/s12929-025-01180-4)
Supplement: Supplementary file 1 — Supplementary material 1. [file 12929_2025_1180_MOESM1_ESM.pptx]

## Slide 1
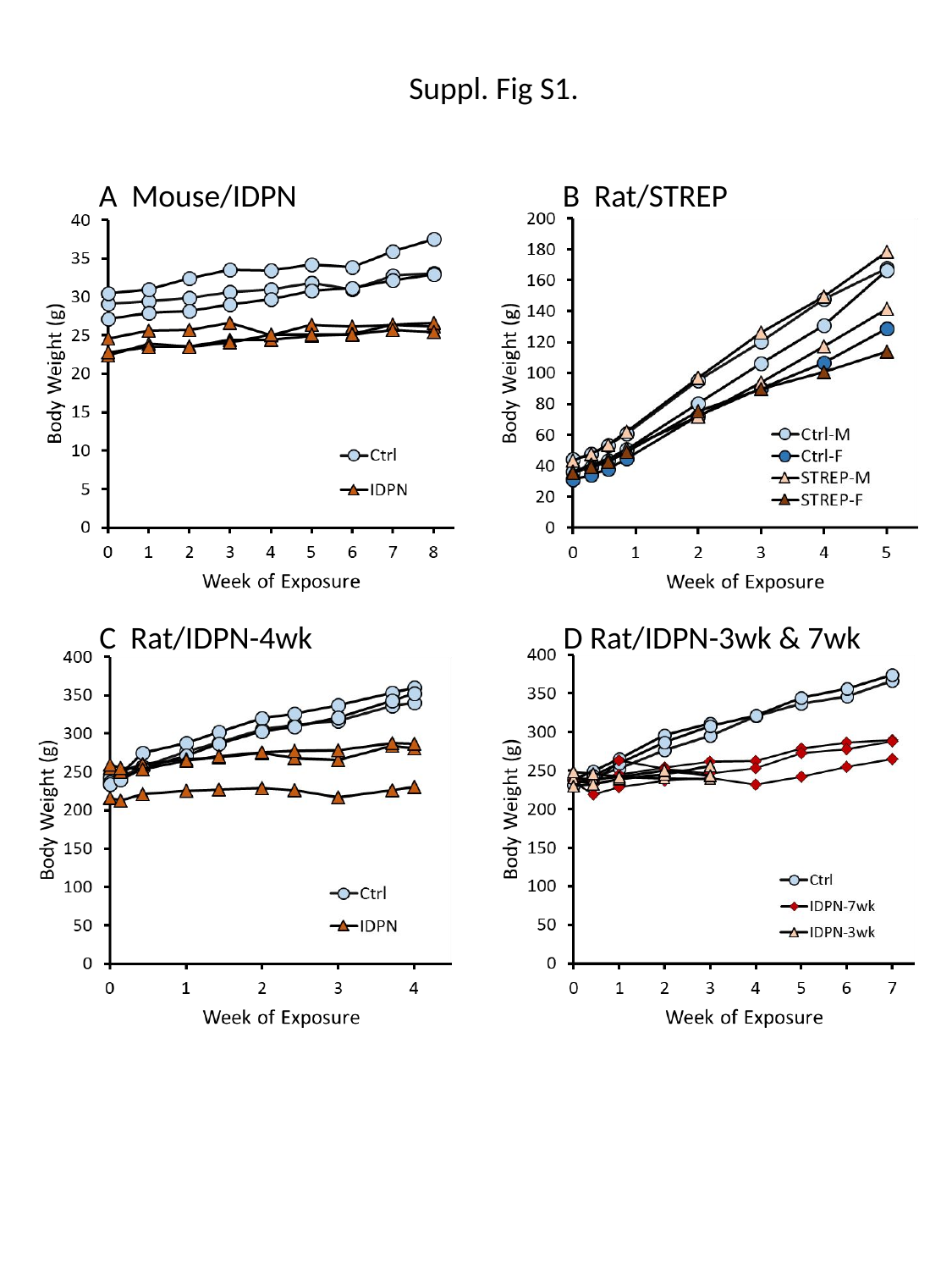

Suppl. Fig S1.
A Mouse/IDPN
B Rat/STREP
C Rat/IDPN-4wk
D Rat/IDPN-3wk & 7wk

## Slide 2
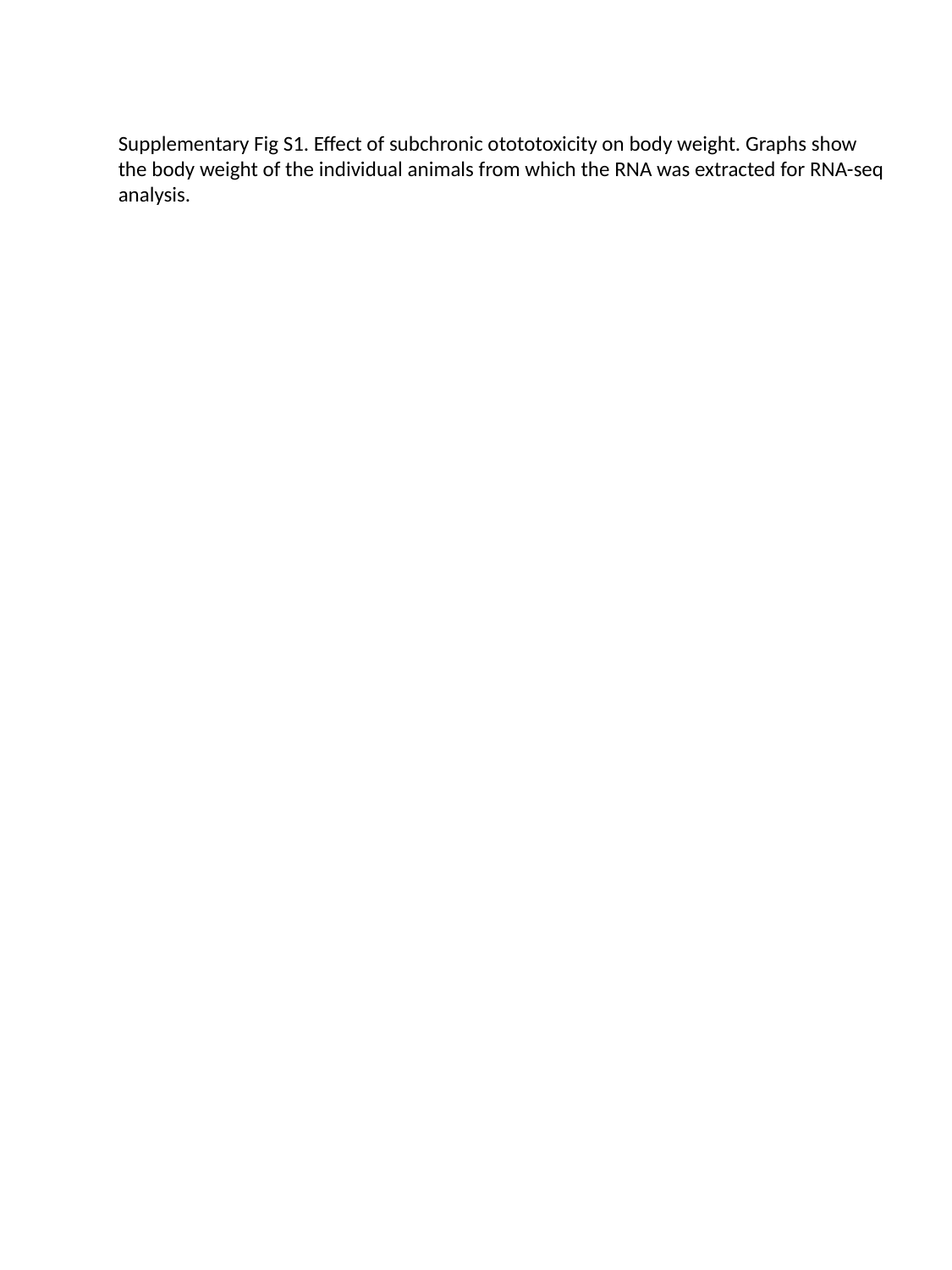

Supplementary Fig S1. Effect of subchronic otototoxicity on body weight. Graphs show the body weight of the individual animals from which the RNA was extracted for RNA-seq analysis.
